# Morphophysiological Differences between the Metapleural Glands of Fungus-Growing and Non–Fungus-Growing Ants (Hymenoptera, Formicidae)

**DOI:** 10.1371/journal.pone.0043570

**Published:** 2012-08-22

**Authors:** Alexsandro Santana Vieira, Odair Correa Bueno, Maria Izabel Camargo-Mathias

**Affiliations:** 1 Departamento de Biologia, Instituto de Biociências, Universidade Estadual Paulista, Rio Claro, São Paulo, Brazil; 2 Centro de Estudos de Insetos Sociais, Instituto de Biociências, Universidade Estadual Paulista, Rio Claro, São Paulo, Brazil; Natural Resources Canada, Canada

## Abstract

The metapleural gland is an organ exclusive to ants. Its main role is to produce secretions that inhibit the proliferation of different types of pathogens. The aim of the present study was to examine the morphophysiological differences between the metapleural gland of 3 non–fungus-growing ants of the tribes Ectatommini, Myrmicini, and Blepharidattini and that of 5 fungus-growing ants from 2 basal and 3 derived attine genera. The metapleural gland of the non–fungus-growing ants and the basal attine ants has fewer secretory cells than that of the derived attine ants (leaf-cutting ants). In addition, the metapleural gland of the latter had more clusters of secretory cells and sieve plates, indicating a greater storage capacity and demand for secretion in these more advanced farming ants. The glands of the derived attine ants also produced higher levels of polysaccharides and acidic lipids than those of Myrmicini, Blepharidattini, and basal attines. Our results confirm morphophysiological differences between the metapleural glands of the derived attines and those of the basal attines and non–fungus-growing ants, suggesting that the metapleural glands of the derived attines (leaf-cutting ants) are more developed in morphology and physiology, with enhanced secretion production (acidic lipids and protein) to protect against the proliferation of unwanted fungi and bacteria in the fungal garden, it is possible that leaf-cutting ants may have evolved more developed metapleural glands in response to stronger pressure from parasites.

## Introduction

Fungus-growing ants (Attini) are especially interesting biological models for comparative studies since they comprise a monophyletic tribe [1] and show distinct evolutionary transitions. One of these transitions involves fungus-growing behavior and another involves a gradual change in colony-size. Eight of the 12 genera (basal attines, such as *Apterostigma* and *Mycetarotes*) form small colonies (<100 individuals) that use several types of detritus, including dead insects and droppings, as substrate to cultivate their fungus [2], [3]. The other 4 genera (derived attines), including *Trachymyrmex* [2], [3], *Sericomyrmex* [4], and leaf-cutting ants, cultivate their fungi on plants and exhibit advanced levels of mutualism, with the fungi producing food bodies rich in proteins. Another derived trait of leaf-cutting ants is multiple mating by queens. The leaf-cutting ants *Acromyrmex* and *Atta* are considered apomorphic among the derived attines and are distinctly different since they have polymorphic workers that exclusively use fresh vegetation as a substrate for their fungi. In addition, their colonies are formed of tens of thousands (*Acromyrmex*) to millions (*Atta*) of individuals [2], [3].

The fungus-growing behavior exhibited by attines shows major evolutionary changes that are unique to ants: (1) The transition to fungus farming by attine ants ∼50 million years ago (MYA) and possibly the need for extra defenses for their fungal crops, a novel challenge that no other ant has ever faced; (2) The transition within attines to rearing a single clade of coevolving gongylidia-producing fungi at the base of higher attines (approximately ∼20 MYA) that may have required more elaborate hygienic defenses when the fungi were more vulnerable and had to be maintained in larger gardens; (3) The transition to active herbivory ∼10 MYA that brought even less diversity into clonal crops, large-scale fungus farming, and possibly novel pathogens that came with fresh leaves [5].

Adaptation against several types of pathogens has been considered one of the main events in the evolution and elaboration of ant societies [6]. The metapleural gland is an organ exclusive to ants and is arranged as a paired structure located at the posterolateral ends of the metathorax [7]. Four hypothesized functions have been proposed for the metapleural gland [8], that the secretion is used for a) species and colony recognition, b) territory marking, c) antisepsis, and d) chemical defense against predators and competitors. Poulsen et al. [9] have presented the best evidence for the importance of the metapleural gland in disease suppression. Sumner et al. [10] demonstrated that socially parasitic ants have smaller metapleural glands and are more susceptible to disease and Hughes et al. [11] inferred a major evolutionary transition in metapleural gland size at the base of the leaf-cutting ant clade. However, both these studies used only the externally visible bulla to assess gland size, and further studies on the internal morphology of the gland are needed to have a complete picture of any transitions in gland function between attine and non-fungus growing ants.

Most recently, studies on the metapleural gland have focused on the chemical composition of its secretions [9], [12]–[15], while few studies have examined its internal morphology [16]–[18]. Fernández-Marín et al. [15] observed that ants exposed to infection with fungal spores cleaned the opening of the metapleural gland more frequently than control ants; this confirmed the importance of this gland in the hygiene of the colonies. In addition, the secretions produced by the metapleural gland of *Acromyrmex octospinosus* inhibit the development in vitro of pathogenic fungi and bacteria to varying degrees [14].

The aim of the present study was to compare morphological differences in the metapleural gland of 3 non–fungus-growing ants of the tribes Ectatommini (*Ectatomma brunneum*), Myrmicini (*Pogonomyrmex naegeli*), and Blepharidattini (*Wasmannia auropunctata*) and 5 fungus-growing ants from 2 basal (*Apterostigma pilosum* and *Mycetarotes parallelus*) and 3 derived lineages (*Trachymyrmex fuscus*, *Acromyrmex coronatus*, and *Atta laevigata*). *Wasmannia auropunctata* was used because its tribe are phylogenetically close to attine ants [7], [19]–[20], while *Ectatomma brunneum* was used because a structural synamorphy of the metapleural gland has been suggested to exist between the Ectatomminae and the Myrmicinae, with external and internal morphologies being very similar among species of these subfamilies [21]. This information is important because studies to data have largely concentrated on evidence from a single species rather than on comparative evidence. In addition, the secretions of the metapleural gland of the different species were examined with specific staining techniques, giving new comparative insights into the chemical composition of the secretions.

## Methods

In the current study, 36 workers of each species were used. The ants differed in size based on head width: *E. brunneum* (2.075±0.132 mm), *P. naegeli* (1.035±0.13 mm), *W. auropunctata* (0.378±0.038 mm), *A. pilosum* (0.716±0.054 mm), *M. parallelus* (0.710±0.017 mm), *T. fuscus* (1.149±0.031 mm), and polymorphic workers of *A. coronatus* (majors: 1.716±0.092 mm, medias 1.360±0.127 mm; minors: 0.934±0.004 mm) and of *A. laevigata* (majors: 3.282±0.335 mm, medias: 2.048±0.172 mm, minors: 0.773±0.016 mm) [22], [23]. These colonies were collected in the Rio Claro campus and maintained in the laboratory of the Center for Studies on Social Insects - UNESP, Rio Claro campus, SP/Brazil.

To obtain the metapleural glands, the legs and heads were removed from the ants in Petri dishes containing saline solution (NaCl, 7.5 g·L^−1^; Na_2_HPO_4_, 2.38 g·L^−1^; and KH_2_PO_2_, 2.72 g·L^−1^) under a Zeiss stereomicroscope with the aid of dissecting forceps and microscissors, leaving only the mesosoma. Glands were fixed with specific fixatives, depending on the technique used (see below). The material was dehydrated in a series of ethanol solutions (70, 80, 90, and 95%) for 15 min each, embedded in resin for 24 h, and transferred to plastic molds previously filled with resin and catalyzer [24]. After polymerization, the material was sectioned at 4 µm with a Leica RM 2145 microtome, hydrated, and placed on glass slides. After the procedures for each technique, dry slides with the sections were mounted using Canada balsam and covered with a cover slip for later observation and photo documentation under a Leica photomicroscope connected to an Intel Pentium 4 computer.

### Histology

#### Harris hematoxylin–aqueous eosin staining [24]

Six metapleural glands of each species were fixed with 4% paraformaldehyde for 24 h. Histological sections were hydrated for 1 min in distilled water and stained with hematoxylin for 10 min. The material was maintained immersed in a cuvette with water for 4 min and rinsed with tap water to allow the reaction to occur. The sections were stained with eosin for 5 min and washed with tap water.

### Histochemistry

#### Periodic Acid Schiff (PAS) reaction [25] for polysaccharide detection and counterstaining with methyl green for RNA detection

Six metapleural glands of each species were fixed with Bouin’s aqueous mixture for 24 h. Histological sections were hydrated for 1 min with distilled water and immediately transferred to a solution of 0.4% periodic acid for 10 min. The material was then washed with distilled water for 1 min and immersed in Schiff’s reagent for 1 h, washed for 30 min with tap water, counterstained with methyl green for 20 s, and rinsed again with water.

#### Bromophenol blue staining to detect total proteins [26]

Six metapleural glands of each species were fixed with 4% paraformaldehyde and 0.9% NaCl in 10% phosphate buffer (0.1 M, pH 7.4) for 24 h. The sections were stained with bromophenol blue for 1 h at room temperature and immersed in an aqueous solution of 0.5% acetic acid for 5 min and then in tertiary butyl alcohol for 5 min.

#### Nile blue staining to detect acidic lipids [27]

Six metapleural glands of each species were removed and fixed with calcium formol for 24 h. Sections were stained with Nile blue for 5 min at 37°C, rinsed in tap water, and immersed in 1% acetic acid for 1 min. After drying, slides with the gland sections were mounted in glycerinated gelatin and covered using a cover slip for observation and photographic documentation.

## Results

### Morphology

**Figure 1:**
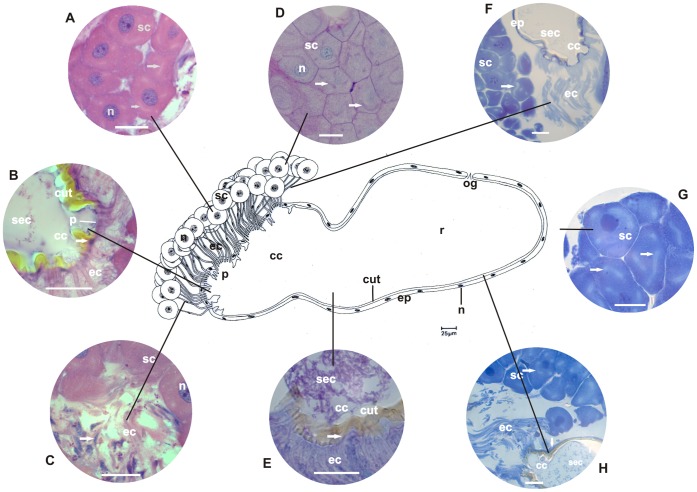
Schematic representation, histology, and histochemistry of the metapleural gland of the basal ant *Ectatomma brunneum*. Secretory cells (**sc**) and their nuclei (**n**), extracytoplasmic portions of canaliculi (**ec**) and their nuclei (**n**), collecting chamber (**cc**), sieve plate (**p**) and reservoir (**r**) with opening (**og**), lined by epithelium (**ep**) and cuticular intima (**cut**) are shown. **A–C:** Histological sections of the metapleural gland stained with hematoxylin and eosin (staining in nucleus and cytoplasm). Details of secretory cells (**sc**) and their nuclei (**n**), intracytoplasmic (**arrow**) and extracytoplasmic portions of canaliculi (**ec**) with nuclei (**n**). Note canaliculi surrounding the nucleus of the secretory cell and arising individually from each cell and opening in the sieve plate (**p**) of the collecting chamber (**cc**) lined by the cuticular intima (**cut**), and the reservoir storing secretion (**sec**). Scale bar = 25 µm. **D–E:** Histological sections of the metapleural gland stained with PAS/methyl green (for detecting polysaccharides and RNA). Details of the secretory cells (**sc**) showing the cytoplasm, nucleus (**n**), intracytoplasmic (**arrow**) and extracytoplasmic (**ec**) canaliculi, collecting chamber (**cc**), and the lining epithelium (**arrow**) and nuclei of cells (**arrow**). Note the presence of secretion (**sec**) containing polysaccharides in the collecting chamber and reservoir. Scale bar = 25 µm. **F:** Histological sections of the metapleural gland stained with bromophenol blue (for detecting total proteins). Details of the secretory cells (**sc**), intracytoplasmic (**arrow**) and extracytoplasmic (**ec**) portions of canaliculi, and collecting chamber (**cc**) lined by epithelium (**ep**). Note the presence of secretion (**sec**) in the collecting chamber. Scale bar = 25 µm. **G–H:** Histological sections of the metapleural gland stained with Nile blue (for detecting acidic lipids). Details of the secretory cells (**sc**), intracytoplasmic (**arrow**) and extracytoplasmic (**ec**) portions of canaliculi, lining epithelium (**arrow**) of the collecting chamber (**cc**), and the reservoir (**r**). Note the presence of secretion (**sec**) containing acidic lipids in the reservoir. Scale bar = 25 µm.

**Figure 2:**
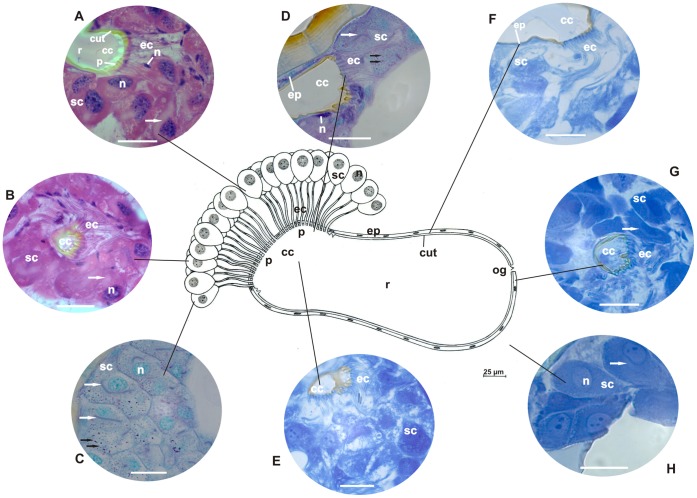
Schematic representation, histology, and histochemistry of the metapleural gland of *Pogonomyrmex naegeli* (Myrmicini). Secretory cells (**sc**) and their nuclei (**n**), extracytoplasmic portions of canaliculi (**ec**) and their nuclei (**n**), collecting chamber (**cc**) and the sieve plate (**p**) and reservoir (**r**) with opening (**og**) lined by epithelium (**ep**), and cuticular intima (**cut**) are shown. **A–B:** Histological sections of the metapleural gland stained with hematoxylin and eosin (staining in nucleus and cytoplasm). Details of secretory cells (**sc**) and nuclei (**n**), intracytoplasmic (**arrow**) and extracytoplasmic portions of canaliculi (**ec**), and their nuclei (**n**). Note the intracytoplasmic portion of canaliculi (**arrow**) surrounding the nucleus of the secretory cells and arising individually from cells to open in the sieve plate (**p**) of the collecting chamber (**cc**) lined by a cuticular intima (**cut**). Scale bar = 25 µm. **C–D:** Histological sections of the metapleural gland stained with PAS/methyl green (for detecting polysaccharides and RNA). Details of the secretory cells (**sc**) showing the cytoplasm, nuclei (**n**), intracytoplasmic (**arrow**) and extracytoplasmic (**ec**) canaliculi, collecting chamber (**cc**), lining epithelium (**ep**), and nuclei (**n**). Note the presence of granules containing polysaccharides (**dark arrow**) in the cytoplasm of the secretory cell. Scale bar = 25 µm. **E–F:** Sections of the metapleural gland stained with bromophenol blue (for detecting total proteins). Details of secretory cells (**sc**), extracytoplasmic portion (**ec**) of canaliculi, and collecting chamber (**cc**) lined by epithelium (**ep**). Scale bar = 25 µm. **G–H:** Histological sections of the metapleural gland stained with Nile blue (for detecting acidic lipids). Details of the secretory cells (**sc**), intracytoplasmic (**arrow**) and extracytoplasmic portions (**ec**) of canaliculi, and collecting chamber (**cc**). Scale bar = 25 µm.

**Figure 3:**
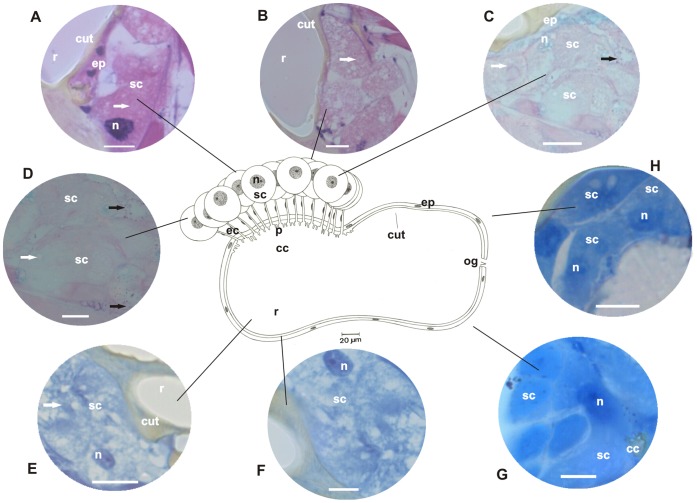
Schematic representation, histology, and histochemistry of the metapleural gland of *Wasmannia auropunctata* (Blepharidattini). Secretory cells (**sc**) and their nuclei (**n**), extracytoplasmic portion of canaliculi (**ec**) and their nuclei (**n**), collecting chamber (**cc**) and the sieve plate (**p**) and reservoir (**r**) with opening (**og**), lined by epithelium (**ep**) and cuticular intima (**cut**) are shown. **A–B:** Histological sections of the metapleural gland stained with hematoxylin and eosin (staining in nucleus and cytoplasm). Details of the secretory cells (**sc**) and nuclei (**n**), intracytoplasmic portion (**arrow**) of canaliculi (**ec**), reservoir (**r**) lined by epithelium (**ep**) and cuticular intima (**cut**). Scale bar = 20 µm. **C–D:** Histological sections of the metapleural gland stained with PAS/methyl green (for detecting polysaccharides and RNA). Details of secretory cells (**sc**) showing the cytoplasm, nucleus (**n**), intracytoplasmic canaliculi (**arrow**), and reservoir (**r**) lined by epithelium (**ep**). Note the presence of few granules containing polysaccharides (**dark arrow**) located in the periphery of the secretory cells. Scale bar = 20 µm. **E–F:** Histological sections of the metapleural gland stained with bromophenol blue (for detecting total proteins). Details of secretory cells (**sc**), nuclei (**n**), intracytoplasmic portion of the canaliculi (**arrow**), and reservoir (**r**) lined by a cuticular intima (**cut**). Scale bar = 20 µm. **G–H:** Histological sections of the metapleural gland stained with Nile blue (for detecting acidic lipids). Details of secretory cells (**sc**) and collecting chamber (**cc**). Scale bar = 20 µm.

**Figure 4:**
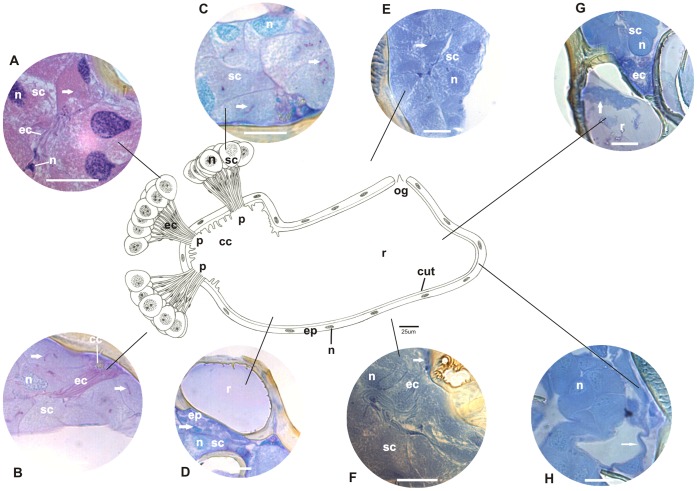
Schematic representation, histology, and histochemistry of the metapleural gland *Apterostigma pilosum* (Attini). Secretory cells (**sc**) and their nuclei (**n**), extracytoplasmic portion of canaliculi (**ec**) and their nuclei (**n**), collecting chamber (**cc**) and the sieve plate (**p**), and reservoir (**r**) with opening (**og**), lined by epithelium (**ep**) and cuticular intima (**cut**) are shown. **A:** Histological sections of the metapleural gland stained with hematoxylin and eosin (staining in nucleus and cytoplasm). Details of secretory cells (**sc**) and nuclei (**n**), intra (**arrow**) and extracytoplasmic portions of canaliculi (**ec**) and their nuclei (**n**). Scale bar = 25 µm. **B–D:** Histological sections of the metapleural gland stained with PAS/methyl green (for detecting polysaccharides and RNA). Details of the secretory cells (**sc**) showing the cytoplasm, nuclei (**n**), cross section of intracytoplasmic canaliculi (**arrow**), longitudinal section of extracytoplasmic canaliculi (**ec**), collecting chamber (**cc**), and reservoir (**r**) lined by epithelium (**ep**) and their nuclei (**arrow**). Scale bar = 25 µm. **E**–**F:** Histological sections of the metapleural gland stained with bromophenol blue (for detecting total proteins). Details of secretory cells (**sc**) and nuclei (**n**), and intracytoplasmic (**arrow**), lumen (**dark arrow**), and extracytoplasmic portions (**ec**) of canaliculi, and collecting chamber (**cc**) lined by epithelium (**arrow**). Scale bar = 25 µm. **G**–**H:** Histological sections of the metapleural gland stained with Nile blue (for detecting acidic lipids). Details of secretory cells (**sc**) and nuclei (**n**), intracytoplasmic (**dark arrow**) and extracytoplasmic portions of canaliculi (**ec**), and reservoir (**r**) lined by epithelium (**arrow**). Note the presence of secretion (**sec**) containing acidic lipids in the reservoir. Scale bar = 25 µm.

**Figure 5:**
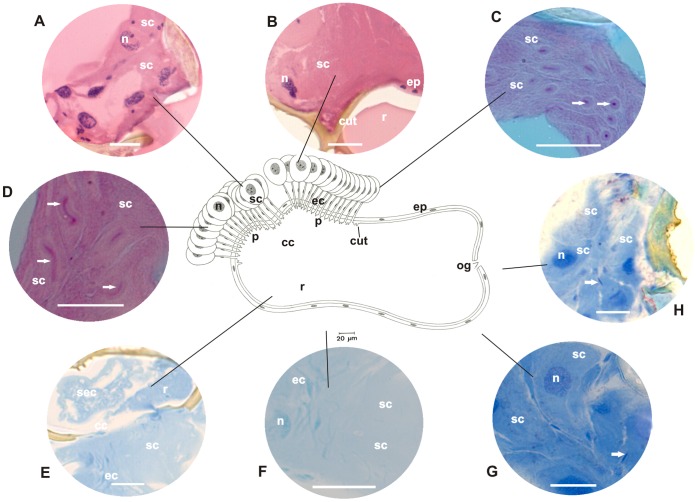
Schematic representation, histology, and histochemistry of the metapleural gland *Mycetarotes parallelus* (Attini). Secretory cells (**sc**), nuclei (**n**), extracytoplasmic portion of canaliculi (**ec**), collecting chamber (**cc**) and the sieve plate (**p**), and reservoir (**r**) with opening (**og**), lined by epithelium (**ep**) and cuticular intima (**cut**) are shown. **A–B:** Histological sections of the metapleural gland stained with hematoxylin and eosin (staining in nucleus and cytoplasm). Details of the secretory cells (**sc**) and nuclei (**n**), reservoir (**r**) lined by epithelium (**ep**) and cuticular intima (**cut**). Scale bar = 20 µm. **C–D:** Histological sections of the metapleural gland stained with PAS/methyl green (for detecting polysaccharides and RNA). Details of the secretory cells (**sc**) and canaliculi intracytoplasmic (**arrow**). Scale bar = 20 µm. **E–F:** Histological sections of the metapleural gland stained with bromophenol blue (for detecting total proteins). Details of the secretory cells (**sc**) and nuclei (**n**), extracytoplasmic canaliculi (**ec**), collecting chamber (**cc**), and reservoir (**r**), lined by a cuticular intima (**cut**). Note the presence of secretion (**sec**) containing proteins in the reservoir. Scale bar = 20 µm. **G–H:** Histological sections of the metapleural gland stained with Nile blue (for detecting acidic lipids). Details of the secretory cells (**sc**) and nuclei (**n**), and extracytoplasmic canaliculi (**arrow**). Scale bar = 20 µm.

**Figure 6:**
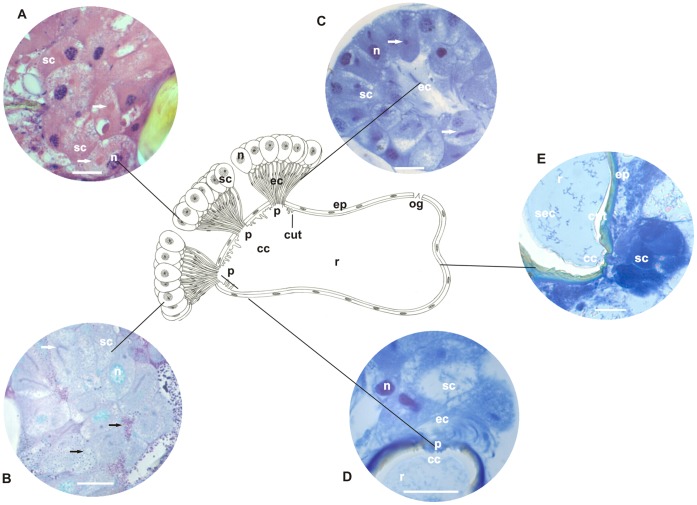
Schematic representation, histology, and histochemistry of the metapleural gland of *Trachymyrmex fuscus* (Attini). Secretory cells (**sc**) and nuclei (**n**), extracytoplasmic portion of canaliculi (**ec**), collecting chamber (**cc**) and the sieve plate (**p**), and reservoir (**r**) with opening (**og**), both lined by epithelium (**ep**) and cuticular intima (**cut**) are shown. **A:** Histological sections of the metapleural gland stained with hematoxylin and eosin (staining in nucleus and cytoplasm) Details of secretory cells (**sc**) and nuclei (**n**), intracytoplasmic portion of canaliculi (**arrow**). Scale bar = 25 µm. **B:** Histological sections of the metapleural gland stained with PAS/methyl green (for detecting polysaccharides and RNA). Details of secretory cells (**sc**) and nuclei (**n**), intracytoplasmic portion of canaliculi (**arrow**). Note the presence of granules containing polysaccharides in the periphery of the secretory cells (**dark arrow**). Scale bar = 25 µm. **C:** Histological sections of the metapleural gland stained with bromophenol blue (for detecting total proteins). Details of secretory cells (**sc**) and nuclei (**n**) and the intracytoplasmic portion of canaliculi (**arrow**). Scale bar = 25 µm. **D:** Histological section of the metapleural gland stained with Nile blue (for detecting acidic lipids). Details of the secretory cells (**sc**) and nuclei (**n**), extracytoplasmic portion of canaliculi (**ec**), collecting chamber (**cc**) and the sieve plate (**p**), and reservoir (**r**) lined by epithelium (**ep**) and cuticular intima (**cut**). Note the presence of secretion (**sec**) containing acidic lipids in the reservoir. Scale bar = 25 µm.

The metapleural glands of *E. brunneum*, *P. naegeli*, *W. auropunctata*, *A. pilosum*, *M. parallelus*, and *T. fuscus* are divided into secretory and storage portions connected by canaliculi (Figures 1, 2, 3, 4, 5, 6). The morphology of these glands has previously been described for *A. laevigata* [22] and *A. coronatus* [23]; thus, in the present study, we only describe the details that differed from those reported in the literature.

The secretory portion of the metapleural glands of the examined ants exhibited clusters of secretory cells that varied in number across species. In *E. brunneum*, 47 secretory cells formed a single cluster; in *P. naegeli*, 30 secretory cells were divided into 2 groups of 15; in *W. auropunctata*, 15 secretory cells were grouped in a single cluster; in *A. pilosum*, 42 cells were distributed in 3 groups of 14; in *M. parallelus*, 30 secretory cells were divided into 2 groups of 15; and in *T. fuscus*, 60 secretory cells were divided into 3 clusters of 20 (Figures 1, 2, 3, 4, 5, 6). Two cell shapes were observed in the secretory portion of the metapleural glands. Round-shaped cells were observed in *E. brunneum* and *W. auropunctata*, while oval-shaped cells were observed in *P. naegeli*, *A. pilosum*, *M. parallelus*, and *T. fuscus* (Figures 1, 2, 3, 4, 5, 6).

In all the examined species, the canaliculus that connected the secretory portion to the storage portion was subdivided into 2 distinct portions: (a) intracytoplasmic and (b) extracytoplasmic portions (Figures 1, 2, 3, 4, 5, 6). In *E. brunneum* and *P. naegeli*, the intracytoplasmic portion surrounded the nucleus of the secretory cell (Figures 1A, F–G; 2A–C, G), while in *W. auropunctata*, *A. pilosum*, *M. parallelus* and *T. fuscus*, the canaliculus meandered in the cytoplasm of the secretory cell (Figures 3A–B; 4C–D;5C–D; 6A–C). The intracytoplasmic canaliculi arose individually from each secretory cell in all the examined species. However, the extracytoplasmic portions of the canaliculi remained either separated or clustered, opening in the sieve plate. Canaliculi were separated in *E. brunneum*, *W. auropunctata*, and *P. naegeli*, and grouped in *A. pilosum*, *M. parallelus*, and *T. fuscus* (Figures 1, 2, 3, 4, 5, 6). The number of canaliculi in each metapleural gland corresponds to the number of secretory cells, since each cell gives rise to 1 canaliculus. In all the examined species, the diameter of the intracytoplasmic portion of the canaliculi was narrower than that of the extracytoplasmic one and was lined by a thin cuticular intima. The extracytoplasmic portion was wider in diameter and had a thicker cuticular intima (Figures 1, 2, 3, 4, 5, 6).

The sieve plate (single or multiple) is located on the wall of the collecting chamber, where the extracytoplasmic canaliculi open. In *E. brunneum* and *W. auropunctata* the sieve plate was a single structure, while 2 sieve plates were observed in *P. naegeli* and *M. parallelus* and 3 plates in *A. pilosum* and *T. fuscus* (Figures 1, 2, 3, 4, 5, 6). Moreover, in *E. brunneum*, *W. auropunctata*, *A. pilosum*, *M. parallelus*, and *T. fuscus* (Figures 1, 3, 4, 5, 6) the cuticular intima of the plate exhibited folds, which were absent in *P. naegeli* (Figure 2). In all the examined species, the collecting chamber and the reservoir were lined by a simple squamous epithelium with cells with flat nuclei and cuticular intima (Figures 1, 2, 3, 4, 5, 6). A summary of the morphohistological results is present in table 1.

**Table 1:** Morphological comparison of the metapleural glands of monomorphic workers of *Ectatomma brunneum, Pogonomyrmex naegeli, Wasmannia auropunctata, Apterostigma pilosum, Mycetarotes parallelus*, and *Trachymyrmex fuscus*, and polymorphic workers of the leaf-cutting ants *Acromyrmex coronatus* and *Atta laevigata*.

	Secretory cells			Intracellular canaliculi	Extracellular canaliculi		Reservoir	
Species	Total number of cells ofeach gland	Number of groupsof secretory cells	Shape of secretorycells	Sinuous or surroundingthe nucleus	Clustered or separate	Canaliculi ineach gland	Number of sieve plates	Absence/presenceof infoldings
***Ectatomma brunneum***	47	single group of cells	round	surrounding the nucleus	separate	47	01	present
***Pogonomyrmex naegeli***	30	2 groups of 15 cells	oval	surrounding the nucleus	separate	30	02	absent
***Wasmannia auropunctata***	15	single group of cells	round	sinuous	separate	15	01	present
***Apterostigma pilosum***	42	3 groups of 14 cells	oval	sinuous	clustered	42	03	present
***Mycetarotes parallelus***	30	2 groups of 15 cells	oval	sinuous	clustered	30	02	present
***Trachymyrmex fuscus***	60	3 groups of 20 cells	oval	sinuous	clustered	60	03	present
* ***Acromyrmex coronatus*** (Vieira et al. 2011)	≥136	8 groups of 17 cells	oval	sinuous	clustered	≥136	≥08	present
* ***Atta laevigata*** (Vieira et al. 2010)	≥140	7 groups of 20 cells	oval	sinuous	clustered	≥140	≥07	present

* already published data, see details in Vieira et al. (2010, 2011) for *Atta laevigata* and *Acromyrmex coronatus* (minor workers).

### Histochemistry

The histochemistry techniques (PAS, bromophenol blue, and Nile blue) applied to the metapleural glands of the workers of *E. brunneum*, *P. naegeli*, *W. auropunctata*, *A. pilosum*, *M. parallelus*, and *T. fuscus* revealed that the secretory cells of all the species contained polysaccharides, proteins, and acidic lipids in the cytoplasm. However, differences in staining intensity were observed.

#### Cells of the secretory portion

Moderate staining of polysaccharides was observed in the cytoplasm of secretory cells of the metapleural gland of *P. naegeli* (Figures 2C–D), *W. auropunctata* (Figures 3C–D), *A. pilosum* (Figures 4B–C), and *M. parallelus* (Figures 5C–D). In *E. brunneum* (Figure 1D) and *T. fuscus* (Figure 6B), the cells were strongly stained for polysaccharides. In *P. naegeli*, stained granules were concentrated near the cell borders (Figures 2C–D). The cytoplasm of the secretory cells of the metapleural gland of *E. brunneum*, *P. naegeli*, *A. pilosum*, and *T. fuscus* was weakly stained with methyl green (Figures 1D; 2C–D; 4B, and C; 6B), while the nuclei were strongly stained (Figures 1D; 2C–D; 4B and C; 6B). The secretory cells were moderately stained for proteins in *W. auropunctata* (Figures 3E–F) and *M. parallelus* (Figures 5E–F), unlike in *E. brunneum* (Figures 1F), *P. naegeli* (Figures 2E–F), *A. pilosum* (Figures 4E and F), and *T. fuscus* (Figures 6C), where the secretory cells were strongly stained for proteins. For all the species, secretory cells were strongly stained for acidic lipids (Figures 1G–H; 2G–H; 3G–H; 4G–H; 5G–H; 6D–E). A summary of the histochemical results is present in table 2.

**Table 2:** Results of the histochemical tests on the metapleural gland of monomorphic workers of *Ectatomma brunneum, Pogonomyrmex naegeli, Wasmannia auropuncata, Apterostigma pilosum, Mycetarotes parallelus*, and *Trachymyrmex fuscus*, and polymorphic workers of the leaf-cutting ants *Acromyrmex coronatus* and *Atta levigata*.

Species	Histochemical tests				Portions of the metapleural gland		
		Targetcompounds	Cytoplasmof secretorycells	Periphery of intracytoplasmic canaliculi	Lumen of intracytoplasmic canaliculi	Lumen of extracytoplasmic canaliculi	Lining epithelial cells
	**PAS**	polysaccharides	+++	+++	++	++	+
	**Methyl Green**	RNA	+	−	−	−	+
***Ectatomma brunneum***	**Bromophenol Blue**	total proteins	+++	−	+	+	+++
	**Nile Blue**	acidic lipids	+++	−	+	+	+++
	**PAS**	polysaccharides	++	+++	+++	++	+
	**Methyl Green**	RNA	+	−	−	−	+
***Pogonomyrmex naegeli***	**Bromophenol Blue**	total proteins	+++	+++	+	+	+
	**Nile Blue**	acidic lipids	+++	+	+	+	+
	**PAS**	polysaccharides	++	+++	+++	++	+
	**Methyl Green**	RNA	−	−	−	−	−
***Wasmannia auropunctata***	**Bromophenol Blue**	total proteins	++	+	++	+	+
	**Nile Blue**	acidic lipids	+++	+++	+++	+++	++
	**PAS**	polysaccharides	++	+++	+	−	+
	**Methyl Green**	RNA	+	−	−	−	++
***Apterostigma pilosum***	**Bromophenol Blue**	total proteins	+++	+++	+	+	++
	**Nile Blue**	acidic lipids	+++	+++	+	+	++
	**PAS**	polysaccharides	++	++	+++	++	+
	**Methyl Green**	RNA	−	−	−	−	−
***Mycetarotes parallelus***	**Bromophenol Blue**	total proteins	++	++	+++	+++	+
	**Nile Blue**	acidic lipids	+++	+++	++	++	+
	**PAS**	polysaccharides	+++	+++	++	++	+
	**Methyl Green**	RNA	+	−	−	−	−
***Trachymyrmex fuscus***	**Bromophenol Blue**	total proteins	+++	+++	+	+	+
	**Nile Blue**	acidic lipids	+++	+++	++	++	++
	**PAS**	polysaccharides	+++	++	−	−	−
	**Methyl Green**	RNA	−	−	−	−	+
* ***Acromyrmex coronatus*** ** (Vieira et al. 2011)**	**Bromophenol Blue**	total proteins	+++	+++	+	+	++
	**Baker**	total lipids	+++	+++	+	+	++
	**PAS**	polysaccharides	+	−	−	−	+
	**Alcian Blue**	glycosaminoglycans	+++	−	+++	+++	++
* ***Atta laevigata*** ** (Vieira et al. 2010)**	**Bromophenol Blue**	total proteins	+++	+++	+	+	+++
	**Nile Blue**	acidic lipids	+++	−	+++	+++	++

* already published data, see details in Vieira et al. (2010, 2011) for *Atta laevigata* and *Acromyrmex coronatus* (minor workers);

(+) weakly positive;

(++) moderately positive;

(+++) strongly positive;

(−) negative.

#### Intracytoplasmic and extracytoplasmic canaliculi

The pericanalicular region of the intracytoplasmic canaliculi of the secretory cells was strongly stained for polysaccharides in *E. brunneum*, *P. naegeli*, *W. auropunctata*, *A. pilosum*, and *T. fuscus* (Figures 1D, 2C–D, 3C, 4B–C, 6B) and moderately stained in *M. parallelus* (Figures 5C–D). The lumen of the intracytoplasmic canaliculi was strongly stained for polysaccharides in *P. naegeli*, *W. auropunctata*, and *M. parallelus* (Figures 2C–D, 3C, 5C–D), moderately stained in *E. brunneum* and *T. fuscus* (Figures 1D, 6B), and weakly stained in *A. pilosum* (Figures 4B–C). The lumen of the extracytoplasmic canaliculi was moderately stained in *E. brunneum*, *P. naegeli*, *W. auropunctata*, *M. parallelus*, and *T. fuscus* (Figure 1D), while it was unstained in *A. pilosum*.

In the pericanalicular region of the intracytoplasmic canaliculi, strong staining for total proteins was observed in *P. naegeli*, *A. pilosum*, and *T. fuscus* (Figures 2F, 4E, 6C), moderate staining was observed in *M. parallelus* (Figure 5F), and weak staining was observed in *W. auropunctata* (Figure 3E). Staining for proteins was not observed in the lumen of *E. brunneum*. The lumen of the intracytoplasmic canaliculi was strongly stained for proteins in *M. parallelus* (Figure 5F), moderately stained in *W. auropunctata* (Figure 3E), and weakly stained in *E. brunneum*, *P. naegeli*, *A. pilosum* and *T. fuscus* (Figures 2F, 4E, 6C). In the extracytoplasmic portion, strong staining for proteins was observed in *M. parallelus* (Figures 5E–F) and weak staining was observed in *E. brunneum*, *P. naegeli*, *W. auropunctata*, *A. pilosum*, and *T. fuscus* (Figures 1F, 2E–F, 4F, 6C).

Furthermore, the pericanalicular region of the intracytoplasmic portion of the canaliculus was strongly stained for acidic lipids in *W. auropunctata*, *A. pilosum*, *M. parallelus*, and *T. fuscus* (Figures 3G, 4H, 5G–H, 6E), weakly stained in *P. naegeli* (Figure 2G), and unstained in *E. brunneum*. The lumen of the intracytoplasmic canaliculi was strongly positive for lipids in *W. auropunctata* (Figure 3G), moderately positive in *M. parallelus* and *T. fuscus* (Figures 5G–H, 6E), and weakly positive in *E. brunneum*, *P. naegeli*, and *A. pilosum* (Figures 2G, 4H). The lumen of the extracytoplasmic canaliculi was strongly positive in *W. auropunctata* (Figure 3G), moderately positive in *M. parallelus* and *T. fuscus* (Figures 5G–H, 6D), and weakly positive in *E. brunneum*, *P. naegeli*, and *A. pilosum* (Figures 1H, 2G, 4G).

#### Storage portion (collecting chamber and reservoir)

The histochemical techniques revealed that the epithelial cells lining the collecting chamber and reservoir were weakly positive for polysaccharides in all the examined species (Figures 1C, 2D, 3C, 4C, 5C, and 6B). However, a secretion consisting of polysaccharides (Figure 1E) was observed in the reservoir of *E. brunneum*. Moderate staining for RNA was observed in the epithelial cells of *A. pilosum*, while weak staining for RNA was observed in *E. brunneum* and *P. naegeli* (Figures 1C, 2D). Strong staining for proteins was detected in the epithelial cells of *E. brunneum* (Figure 1F), moderate staining in *A. pilosum* (Figure 4F), and weak staining in *P. naegeli*, *W. auropunctata*, *M. parallelus*, and *T. fuscus* (Figures 2F, 3E). The epithelial cells were also strongly stained for lipids in *E. brunneum* (Figure 1H), moderately stained in *W. auropunctata*, *A. pilosum*, and *T. fuscus* (Figures 4H, 6E), and weakly stained in *P. naegeli* and *M. parallelus* (Figures 2G, 5H). A summary of the histochemical results is present in table 2.

## Discussion

The results of the present study on the metapleural gland of ants from the groups Ectatommini, Myrmicini, Blepharidattini, and Attini (basal and derived attines) confirmed the presence of secretory and storage portions connected by canaliculi, supporting the results obtained for the leaf-cutting ants *Atta bisphaerica* and *Atta sexdens rubropilosa* [28], *Acromyrmex octospinosus* [16], *Acromyrmex subterraneus* [18], as well as *Atta laevigata* and *Acromyrmex coronatus* [22], [23].

The non–fungus-growing ants (Ectatommini, Myrmicini, Blepharidattini) had fewer secretory cells than the leaf-cutting ants. Blepharidattini ants (*W. auropunctata*) may be an evolutionary lineage that experienced a reduction in the number of secretory cells as a result of the use of antimicrobial venom, such as in *Polyrhachis dives* [29], or a reduction in parasite pressure. Some ants, such as some lineages of *Camponotus* are devoid of metapleural glands, possibly because of a lower susceptibility to pathogens as they build arboreal nests [30], [31]. However, Walker and Hughes [32] demonstrated that arboreal species were not less resistant to *Metarhizium anisopliae* than ground-dwelling species, and the species that inhabited both arboreal and ground habitats had the greatest resistance. The Myrmicini (*P. naegeli*) and Ectatommini (*E. brunneum*) are non–fungus-growers that build nests in the ground, and their metapleural gland may be physiologically modified to undertake other roles. For example, the metapleural gland secretion in some myrmicine ants has been suggested to be used for marking territories or nest entrances [33]–[36].

On the basis of the literature, attines are phylogenetically divided into basal and derived ant lineages [37]. However, results of the current study indicates that the number of secretory cells in the metapleural gland supports the phylogenetic description by Brady et al. [37], by demonstrating that basal attines (*A. pilosum* and *M. parallelus*) have fewer secretory cells, an intermediary number between those of the derived attine *T. fuscus.* In addition, the leaf cutting ants *A. laevigata* and *A. coronatus* have more secretory cells, indicating higher secretory capacity. The primary role of the metapleural gland is to produce antimicrobial compounds to protect the ants and against parasites. In the case of attine ants, it has been suggested to also protect the mutualistic fungal crop against parasites and to support the growth of mutualistic actinomycete bacteria on the ant cuticle [38]–[39], [8].

The current study demonstrated that in Ectatommini, Blepharidattini, Myrmicini, and the basal attine *M. parallelus*, the secretory cells are arranged in one or two groups, while in the basal attine *A. pilosum* and the derived attine *T. fuscus*, the secretory cells are arranged in 3 groups. In the derived attines *Atta laevigata* and *Acromyrmex coronatus* [22], [23], several groups of secretory cells (7 or 8) were found, suggesting that the number of secretory cells is associated with the evolutionary lineage of the tribe. Therefore, derived attines have more groups of secretory cells, and, as a consequence, they have a greater production of secretion.

The shape of the secretory cells may also indicate the secretory capacity of the metapleural gland in ants. For example, cells are round-shaped in Ectatommini and Blepharidattini and oval-shaped in Myrmicini and Attini. This oval morphology has also been observed in the attines *Atta bisphaerica*, *Atta sexdens rubropilosa* [28], *Acromyrmex octospinosus* [16], *Acromyrmex subterraneus* [18], *Atta laevigata*, and *Acromyrmex coronatus* [22], [23], and the round-shaped cells have been observed in *Diacamma rugosum* and *Diacamma vagans* [40]. According to Junqueira and Carneiro [41], cells taller rather than shorter (oval) have the physiology typical of secretory cells, suggesting that oval-shaped secretory cells of the metapleural gland of attines produce secretions more actively than the metapleural glands of Ectatommini and Blepharidattini.

In the present study, Ectatommini, Myrmicini, and Attini (basal and derived ants) exhibited metapleural glands with secretory cells that stained strongly for the presence of proteins, while in Blepharidattini, the secretory cells were moderately stained for proteins. Ultrastructural analysis has also revealed protein compounds in the secretion of cells of the metapleural gland on the basis of the presence of abundant rough endoplasmic reticulum [38], [42]. This suggests that these glands are also capable of synthesizing protein compounds that stain intensely, but in Blepharidattini, the proteins stain to a lesser extent. These protein compounds probably contribute to most of the antibacterial and antifungal properties, but further investigation of their full composition and function is required.

Staining for the presence of acidic lipids and polysaccharides was observed in the secretory cells of all the examined groups, similar to that previously observed in *Atta laevigata* and *Acromyrmex coronatus* [22], [23]. However, in *E. brunneum*, *T. fuscus*, and leaf-cutting ants [22]–[23], the secretory cells were strongly stained for polysaccharides. Gusmão [28], who studied *A. bisphaerica* and *A. sexdens rubropilosa*, and Vieira et al. [42], who studied *A. laevigata*, observed a significant amount of smooth endoplasmic reticulum. These results demonstrate that derived attines (leaf-cutting ants) and fungus-growers synthesize more polysaccharides and acidic lipids than the non–fungus-growing ants (Ectatommini, Myrmicini, and Blepharidattini), which may provide antibacterial and antifungal properties (protein) or energy reserves (polysaccharides) for future use.

The canaliculi connect the secretory portion to the storage portion of the metapleural gland in ants and are divided into intracytoplasmic and extracytoplasmic portions [43]. The intracytoplasmic portion in Ectatommini and Myrmicini surrounds the nuclei, whereas in Blepharidattini and Attini (basal and derived attines, *T. fuscus*), this portion meanders around the nucleus, possibly to increase the surface area for the collection of elements synthesized by the secretory cells. Our results concur with those of previous studies in which the intracytoplasmic portion in the leaf-cutting ants *Atta laevigata* and *Acromyrmex coronatus* was observed to meander around the nucleus [22], [23]. Likewise, according to Vieira et al. [42], the intracytoplasmic portion of the canaliculi of the metapleural gland of *A. laevigata* has microvilli to increase the surface area, consequently collecting more secretions produced by the secretory cells. These findings are consistent with a higher demand for secretions in these more advanced farming ants.

The secretions produced by the secretory cells of the metapleural gland in all the examined groups, as well as in *Atta laevigata* and *Acromyrmex coronatus* [22], [23], differed from those found in the lumen (inside canaliculi) of the extracytoplasmic portion of the canaliculi. Polysaccharides, proteins, and acidic lipids were strongly stained in the cytoplasm and weakly or moderately stained in the lumen of the extracytoplasmic portion, indicating that in addition to collecting and transporting the secretions, these structures also modify their composition.

The number of canaliculi in each metapleural gland corresponds to the number of secretory cells, since each cell gives rise to 1 canaliculus. In Ectatommini, Blepharidattini, Myrmicini, and Attini (basal), fewer canaliculi were observed than those in the derived attines. Furthermore, the sieve plate, located in the collecting chamber of the storage portion of the metapleural gland, varied in number, depending on the group. In Ectatommini, Blepharidattini, Myrmicini, and Attini (basal), fewer plates were observed than those in the derived attines. A greater number of plates have also been observed in the derived attines *Acromyrmex octospinosus* [16], *Atta sexdens* [30], *Atta bisphaerica*, and *Atta sexdens rubropilosa* [18]. In the ponerine ant *D. vagans* and the doryline (*Dorylus* spp. [44]) and dolichoderine ants (*Dolichoderus quadripunctatus*, *Linepithema humile*, *Tapinoma erraticum* [45]), a single large sieve plate was observed, suggesting that derived attines have more plates in order to receive more extracytoplasmic canaliculi because of a larger number of secretory cells. However, our results showed that the closest outgroup, *W. auropunctata*, had the lowest number of plates and canaliculi, suggesting that the disease pressure in this species is not severe. From this species onwards, the number of plates and canaliculi increases throughout the attines. In addition, our results highlight that smaller increases in plate number in the more distant outgroups are appropriate: *Ectatomma* as a predator/scavenger [46] has probably rather dirty (infectious) food and *Pogonomyrmex* has independently evolved multiple-queen mating [47], quite possibly also in response to higher disease pressure.

The cuticular intima of the collecting chamber of the metapleural glands had infoldings in the area of the sieve plates in Ectatommini, Blepharidattini, and Attini (basal and derived), suggesting that these structures may direct the flow of secretions towards the reservoir (storage portion). A narrow ridge has been observed in the reservoir wall of *A. sexdens*, and it is thought to guide secretions from the collecting chamber toward the opening of the metapleural gland [48]. Nevertheless, the reservoir and the collecting chamber were lined by a simple squamous epithelium in all the tribes examined in this study. According to Vieira et al. [22], lining may not be the only role of these cells, which possibly secrete compounds of the cuticular intima, a protective structure of the epithelial wall, to protect against its own secretion and/or from possible contaminants that could minimize or inactivate its action.

Three hypotheses have been proposed to explain the presence of more secretory cells, clusters of secretory cells, and sieve plates in leaf-cutting ants than in non–fungus-growing ants (Ectatommini, Myrmicini, and Blepharidattini) and the basal attines. First, the queens of leaf-cutting ants mate with multiple males, whereas those of other attines mate only once [49]. Hamilton [50], Schmid-Hempel [51], and Hughes and Boomsma [52] suggested that polyandrous queens of social insects may have evolved because, the more genetically diverse colonies they produce are more resistant to parasites. According to Hughes et al. [11], both polyandry and large metapleural gland reservoirs may have evolved in response to stronger pressure from parasites Second, Hughes et al. [11] suggested that leaf-cutting ants may be able to invest more in resistance since they use fresh vegetation as a substrate for their fungus, which in turn provides more resources for them to use. Third, leaf-cutting ant workers are polymorphic and include individuals specialized in cutting leaves, unlike other lower attines [11]. However, our results did not show an abrupt evolutionary transition in the secretory defense in fungus-growing ants, in which the diameter of the bulla is significantly enhanced only at the final transition to leaf-cutting ants, as reported by Hughes et al. [11]. Our findings indicated that glandular capacity was already partially enhanced in *Trachymyrmex*, suggesting that secretion capacity increased before reservoir capacity.

In conclusion, morphophysiological differences showed that the derived attines (leaf-cutting ants) had more secretory cells, cluster cells, and sieve plates, as well as higher levels of polysaccharides and acidic lipids, than Ectatommini, Myrmicini, Blepharidattini, and basal Attini. The metapleural glands are also capable of synthesizing protein compounds. This suggests that the metapleural glands of derived attines are more developed in morphology and physiology, with enhanced secretion production (acidic lipids and protein) to protect against the proliferation of unwanted fungi and bacteria in the fungal garden. When compared with basal attines and non–fungus-growing ants, it is probable that leaf-cutting ants may have evolved more developed metapleural glands in response to stronger pressure from parasites.
